# Late Quaternary loss of genetic diversity in muskox (*Ovibos*)

**DOI:** 10.1186/1471-2148-5-49

**Published:** 2005-10-06

**Authors:** Ross DE MacPhee, Alexei N Tikhonov, Dick Mol, Alex D Greenwood

**Affiliations:** 1 Division of Vertebrate Zoology, American Museum of Natural History, New York, New York 10024, USA; 2 Laboratory of Mammals, Zoological Institute, Russian Academy of Sciences, Universitetskaya nab. 1, 199034, St. Petersburg, Russia; 3 Cerpolex/Mammuthus, Gudumholm 41, NL-2133 HG Hoofddorp, Netherlands; 4 GSF-National Research Center for Environment and Health, Institute of Molecular Virology, Ingolstaedter Landstr.1, 85764 Neuherberg, Germany

## Abstract

**Background:**

The modern wildherd of the tundra muskox (*Ovibos moschatus*) is native only to the New World (northern North America and Greenland), and its genetic diversity is notably low. However, like several other megafaunal mammals, muskoxen enjoyed a holarctic distribution during the late Pleistocene. To investigate whether collapse in range and loss of diversity might be correlated, we collected mitochondrial sequence data (hypervariable region and cytochrome *b*) from muskox fossil material recovered from localities in northeastern Asia and the Arctic Archipelago of northern North America, dating from late Pleistocene to late Holocene, and compared our results to existing databases for modern muskoxen.

**Results:**

Two classes of haplotypes were detected in the fossil material. "Surviving haplotypes" (SHs), closely similar or identical to haplotypes found in modern muskoxen and ranging in age from ~22,000 to ~160 yrbp, were found in all New World samples as well as some samples from northeastern Asia. "Extinct haplotypes" (EHs), dating between ~44,000 and ~18,000 yrbp, were found only in material from the Taimyr Peninsula and New Siberian Islands in northeastern Asia. EHs were not found in the Holocene muskoxen specimens available for this study, nor have they been found in other studies of extant muskox populations.

**Conclusion:**

We provisionally interpret this evidence as showing that genetic variability was reduced in muskoxen after the Last Glacial Maximum but before the mid-Holocene, or roughly within the interval 18,000-4,000 yrbp. Narrowing this gap further will require the recovery of more fossils and additional genetic information from this interval.

## Background

**Figure 1:**
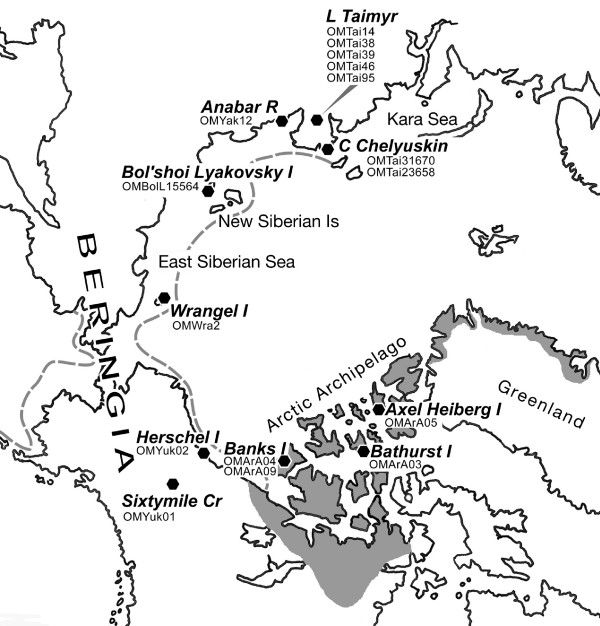
**Sketch map of Holarctic region in polar view, noting places mentioned in text and specimens sampled for mtDNA analysis**. Dark gray area, muskox range ca. AD 1870 [6]; C, cape; Cr, creek; I, island; L, lake; R, river. During the late Pleistocene, *Ovibos *ranged from western Europe to North America via Beringia. In the Old World, presence of muskox fossils on islands north of mainland Asia presumably indicates that *Ovibos *was able to extend its range during full glacial times to the subaerial parts of the continental shelf (dashed line). In the New World, in addition to central Alaska, unglaciated areas in the western Arctic Archipelago may have continuously supported muskoxen also, although the finite ^14^C fossil record for these islands does not begin until about 6800 yrbp [40].

**Figure 2:**
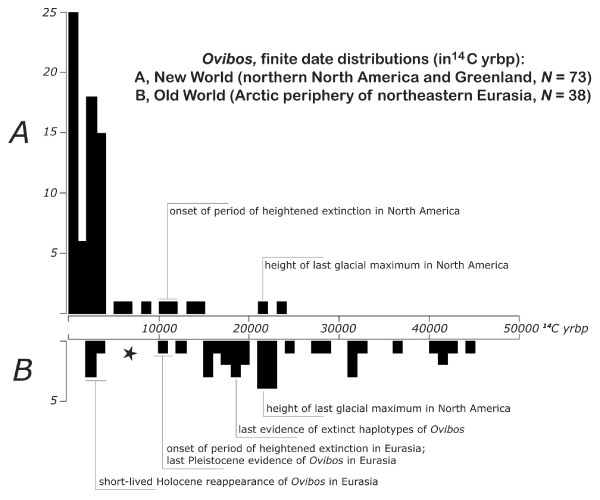
**Finite ^14^C date distributions for fossil specimens of *Ovibos***. (*A*) northern North America and Greenland (*N *= 72; *X *= 3222 yr, *SD *= 4313 yr) and (*B*) Arctic periphery of Asia east of Kara Sea (*N *= 38; *X *= 22,522 yr, *SD *= 11,145 yr), collated from the literature [32,40,41]. In addition to a notable difference in dating intensity, with many more published ^14^C dates being available for New World *Ovibos*, there is also a considerable difference in sample statistics. Skewed date distribution for New World *Ovibos *reflects long-term effect of former icecaps on amount of habitable area [42]. In northeastern Asia, where only small, local (cordilleran) icecaps formed, potential range was less affected and *Ovibos *was presumably continuously present during the last 40–50,000 years. Star in B denotes 7,000 yr gap between "last" Pleistocene and "first" Holocene dated occurrences of *Ovibos *in Asia. Slight offset in "period of heightened extinction" in New World vs. Old World, amounting to approximately 500–1000 yr, reflects a moderate difference in "last" occurrence dates for mainland mammoths in the two areas [32].

The large-mammal (megafaunal) diversity of North America and Eurasia was considerably reduced by extinctions that occurred around the time of the Pleistocene/Holocene transition (i.e., ca. 10,000 years ago) [1]. Interestingly, some megafaunal species (e.g., woolly mammoth, *Mammuthus primigenius*) became extinct at this time in North American and Eurasian continental areas but survived on nearby islands for thousands of years thereafter [2,3]. Some other species whose ranges likewise straddled the Old and New Worlds became extinct on one continent, but not the other, at the end of the Pleistocene (e.g., horse, *Equus caballus*; saiga, *Saiga tatarica*). A third pattern, involving a much lengthier decline, characterizes *Ovibos moschatus *(tundra muskox), the subject of this paper (fig. 1, 2).

According to the available fossil record, the lineage terminally represented by *O. moschatus *arose in Eurasia but entered North America at least as early as the early Pleistocene [4-6]. At roughly the same time several other closely-related Asian ovibovine lineages (e.g., *Soergelia*, *Praeovibos*) also entered North America, where they evidently prospered before disappearing later in the Pleistocene. By the start of the Holocene, muskox diversity had been reduced to surviving *O. moschatus*; the species is now native only to the islands of the Arctic Archipelago and Greenland (fig. 1), if late 20th-century efforts to reintroduce muskoxen into various parts of their ancient range are ignored [6].

This lengthy history of muskox diversification in Quaternary Holarctica stands in sharp contrast to the extremely limited genetic diversity characterizing modern *O. moschatus*. Groves' [7,8] analysis of sequence data for cytochrome *b *(cyt *b*) and the mitochondrial hypervariable region in 37 individuals of *O. moschatus *from various localities in Alaska, Canada, and Greenland revealed only marginal genetic differentiation within extant populations. Groves [7] identified a total of 8 hypervariable region haplotypes, but these differed individually by only 1 or 2 nucleotide substitutions in approximately two-thirds of the cases, for a maximum of 9 differences – a total of 1.3% variation across 697 bp (base pairs), including insertions and deletions. Extreme genetic homogeneity is also indicated by microsatellite analysis [[9], but see also [10]] and the nuclear Mhc *DRB*3 gene [11]. As several different populations were sampled for these studies, in the absence of any other plausible explanation it seems reasonable to conclude that living muskoxen simply lack genetic diversity. The nuclear and mitochondrial data therefore complement each other and imply that muskoxen either passed through a long period of very small effective population sizes, or experienced one or more genetic bottlenecks in very recent times. The investigation reported here was prompted by the possibility that ancient DNA (aDNA) analyses might shed some light on the complex history of extirpation, replacement, and differential survival that have evidently affected muskoxen populations during the late Quaternary. Ancient DNA analysis has been successfully applied to late Pleistocene and Holocene (including the early 20^th ^century) remains by various groups [e.g., [12-22]]. Because of existing uncertainty concerning species boundaries among some late Quaternary members of tribe Ovibovini, the terms "ovibovine" and "muskox" will be used to make general reference to such taxa, without endorsing any particular view as to their taxonomic distinctiveness or geographical distribution.

## Results

**Figure 3:**
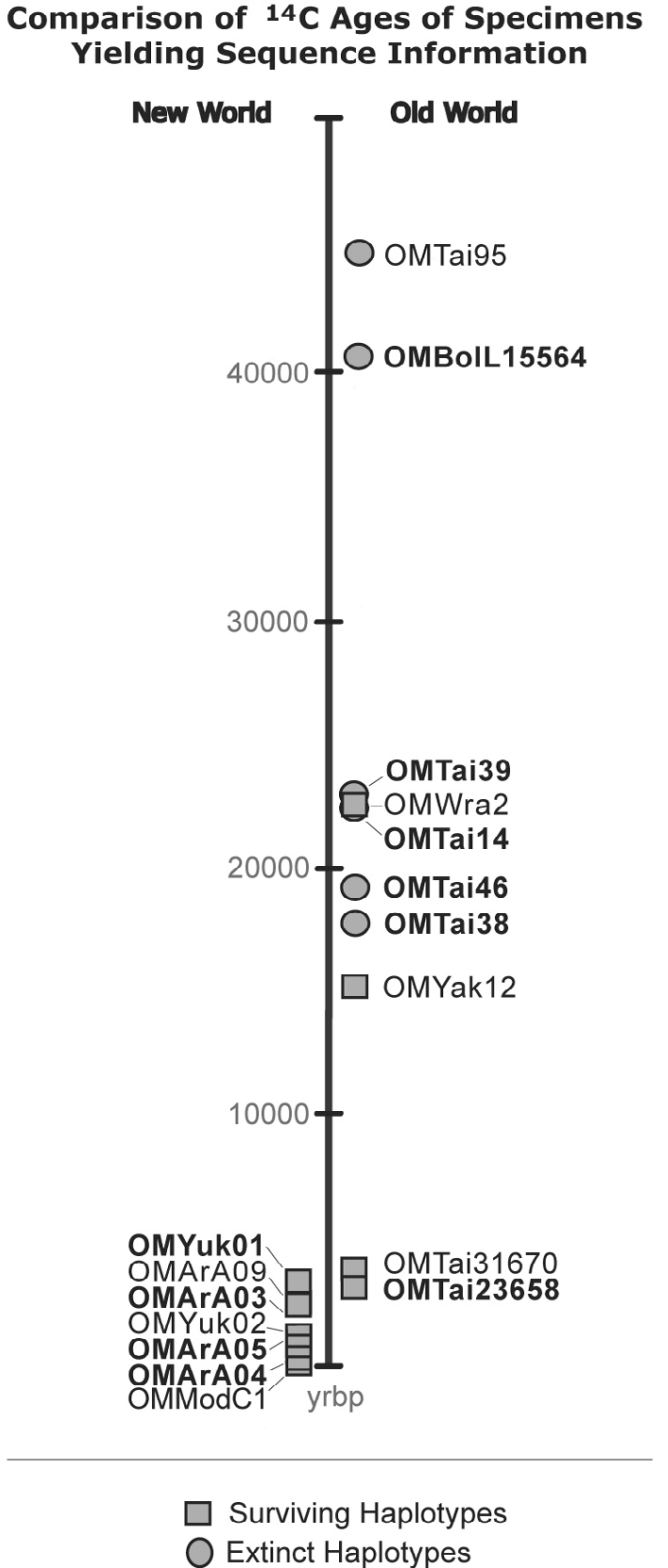
**Timeline showing distribution of specimens yielding sequence information**. Symbols indicate whether a given specimen exhibited an "extinct" (EH) or a "surviving" (SH) haplotype. Specimens yielding substantial sequence data are in bold. SHs (i.e., haplotypes represented in extant *O. moschatus*) were found in all New World samples tested, as well as some Old World samples. EHs have been found so far only in Asian samples.

Our results permit empirical recognition of two haplotype groupings within late Quaternary muskoxen. The first consists of haplotypes recovered from Pleistocene samples collected at various localities in northeastern Asia, including islands north of the mainland (fig. 1, 3). These haplotypes, which differ from all New World material so far examined as well as from the few known Asian specimens that are of Holocene age, appear to have completely disappeared and may therefore be grouped as "EHs" (extinct haplotypes). All other known haplotypes, which include those found in all living muskoxen, are very similar *inter se *and thus comprise a second grouping, "SHs" (surviving haplotypes).

### Sequence verification

A total of 9 fossil samples yielded reproducible sequence covering cyt *b *and the hypervariable region of the mtDNA genome; with the exception of OMTai14, each fragment from these samples was successfully amplified at least twice (additional fig. 1, 2, 3; table 1). One other sample (OMBolL15564) yielded consistent results for cyt *b *but the HV sequence could not be accurately scored (table 1, additional fig. 4). Sequences were then determined via multiple clones produced from each PCR product, a standard aDNA protocol for insuring correct sequence determination [12,22] (additional fig. 1, 2, 3). In addition, several samples were independently extracted by different individuals, amplified, and cloned. Clones were sequenced in one or another of the three separate laboratories involved in this investigation (AMNH, New York; Medigenomix GmbH, Munich; SR&D GmbH, Martinsried).

**Table 1:** Information on bone samples utilized in aDNA studies, with 14C age estimates

**Haplotype Grouping**^*a*^	**aDNA Sample Designation**	**Specimen Institutional Number**^*b*^	**Age**^*c*^	**Dating Lab Number**^*d*^	**Cyt *b *Reps**^*e*^	**HV Reps**^*e*^	*Specimen*	*Locality*
EH group	*OMTai95*	*CME 2000/125*	*44,760 ± 1700*	*B177044*	*1x/1x*	*1x/0x*	*skull*	*Lake Taimyr, Taimyr Pen.*
	*OMBolL15564*	*ZIN 15564*	*40,270 ± 450*	*B193436*	*2x/2x*	*4x/3x*	*skull*	*Bol'shoi Lyakhovski I., New Siberian Is.*
	*OMTai39*	*CME 2000/002*	*22,550 ± 100*	*B148652*	*2x/2x*	*2x/2x*	*metapodial*	*Lake Taimyr, Taimyr Pen.*
	*OMTai14*	*CME unnumb.*	*22,360 ± 80*	*B156194*	*1x/1x*	*2x/2x*	*metapodial*	*Lake Taimyr, Taimyr Pen.*
	*OMTai46*	*CME 2000/102*	*19.230 ± 80*	*B148627*	*2x/2x*	*2x/3x*	*atlas*	*Lake Taimyr, Taimyr Pen.*
	*OMTai38*	*CME 2000/126*	*18,310 ± 70*	*B148628*	*2x/2x*	*2x/2x*	*horn sheath*	*Lake Taimyr, Taimyr Pen.*
	
SH group	*OMWra2*	*ZIN unnumb.*	*22,280 ± 120*	*B193434*	*0x/2x*	*0x/2x*	*unspecified*	*Wrangel I., Chuckchi Sea*
	*OMYak12*	*ZIN unnumb.*	*15,610 ± 80*	*B193435*	*0x/1x*	*0x/2x*	*pelvis*	*Anabar R., Yakutia*
	OMTai31670	ZIN 31670	3,790 ± 80^*f*^	LU 52	0x/0x	1x/2x	skull	Cape Chelyuskin, Taimyr Pen.
	OMYuk01	CMN 36137	3,280 ± 90^*h*^	I10985	2x/2x	2x/2x	skull	Sixtymile Creek, Yukon
	OMTai23658	ZIN 23658	2,970 ± 40^*g*^	B157714	2x/2x	2x/5x	skull	Cape Chelyuskin, Taimyr Pen.
	OMArA09	CMN 17674	2,520 ± 100^*h*^	I3577	0x/0x	0x/1x	horncore	Banks I., Arctic Archipelago
	OMArA03	CMN 34515	2,400 ± 40	B179009	2x/2x	2x/2x	skull	Bathurst I., Arctic Archipelago
	OMYuk02	CMN 17678	1,020 ± 40	B179008	0x/1x	0x/1x	horn core	Herschel I., Yukon
	OMArA05	CMN 49941	700 ± 70^*h*^	TO2703	2x/2x	2x/2x	humerus	Axel Heiberg I., Arctic Archipelago
	OMArA04	CMN 17676	160 ± 40	B195369	2x/2x	2x/2x	horncore	Banks I., Arctic Archipelago
	OMModC1	male zoo animal	(modern)	-	1x/1x	-	hair	Tierpark Hellabrunn, Munich

Notes. – ^*a*^EH, extinct haplotype group; SH, surviving haplotype group (see text). For clarity, in table EH and SH samples are separated by a horizontal line; italics distinguish Pleistocene samples.

^*b*^Institutional abbreviations: CME, Cerpolex/Mammuthus Expeditions, Khatanga; CMN, Canadian Museum of Nature, Ottawa; ZIN, Zoological Institute, Russian Academy of Sciences, St. Petersburg.

^*c*^In ^14^C yrbp ± 1 σ (not δ^13^C corrected).

^*d*^Radiocarbon dating lab abbreviations: B, Beta Analytic, Inc.; TO, IsoTrace Laboratories, University of Toronto; I, Isotopes (Teledyne); LU, Geological Institute, Leningrad State University.

^*e*^Entries indicate whether sequence was obtained for each of 2 overlapping fragments; number indicates number of times bases in fragments were separately covered by PCRs (e.g., 2x/2x indicates each overlapping fragment was amplified and sequenced twice independently).

^*f*^C.R. Harington (pers. commun.), correcting date of "3800 ± 200 yrbp" reported by [43].

^*g*^Also dated by Harington [44], who reported an age estimate of 2910 ± 95 yrbp.

^*h*^As reported by Harington [44].

For example, to determine the cyt *b *sequence for sample OMTai38, two PCRs were performed for the first fragment and second overlapping fragment and five clones sequenced for each PCR product (additional fig. 2). The sequences summarized in tables 3, 4, 5 are thus the consensus of independent replications and multiple-clone sequences for each individual PCR fragment (with a few exceptions), and are unlikely to be laboratory artifacts (additional fig. 1, 2, 3). For this particular sample, all clones from the 3' fragments were identical with one exception: in clone 1.2, there is a C-T transition which most likely represents a polymerase error. In the 5' fragment, all five clones of the first PCR were identical. In the second fragment three classes of sequence were obtained, represented by clones 2.3 and 2.4; these were identical to the clones from the first PCR and matched the 3' fragment in the overlap. The second and third class of sequences (2.1, 2.2, and 2.5) were different from all other clones obtained in the study, unique to the second PCR amplification, and did not match the 3' fragment in the overlap. Blast searching the sequence revealed perfect or near perfect homology for both divergent sequences with other bovid species such as the domestic sheep, *Ovis aries*. These likely represent nuclear mitochondrial DNA sequences (Numts) and thus the sequence represented in the two independent fragments was scored as the bona fide mtDNA sequence, the consensus of which was used for further study.

**Table 3:** DNA sequence variation in the cyt *b *gene

**Source**	**Sample**	**324**	**346**	**366**	**425**	**436**
Modern	SH-OMMod01	T	A	T	A	C
	SH-OMMod02	.	.	.	.	.
	SH-OMMod03	.	.	.	.	.
	SH-OMMod04	.	.	.	.	.
	SH-OMMod05	.	.	.	.	.
	SH-OMModCl	.	.	.	.	.
New World	SH-OMYuk01	.	.	.	.	.
	SH-OMArA03	.	.	.	.	.
	SH-OMArA04	.	.	.	.	.
	SH-OMArA05	.	.	.	.	.
Old World	SH-Wra02	nd	nd	.	.	.
	SH-OM23658	.	.	.	.	.
	*EH-OMTai14*	*C*	*C*	*C*	.	*T*
	*EH-OMTai38*	*C*	*C*	*C*	.	.
	*EH-OMTai39*	*C*	*C*	*C*	.	*T*
	*EH-OMTai46*	*C*	*C*	*C*	.	.

Notes. – Sample names and symbols as in table 2. Positions numbered from first base of ATG start codon of cyt *b*. Extinct haplotypes italicized; "nd", not determined.

**Table 4:** Sequence comparisons between Holocene and Pleistocene samples

	**Cyt *b***		**HV region**	
	**Holocene**	**Pleistocene**	**Holocene**	**Pleistocene**

Number of individuals	43	5	44	4
Number of haplotypes	1	3	4	2
Haplotype diversity	0	0.667 ± 0.204	0.215 ± 0.081	0.667 ± 0.204
Nucleotide diversity	0	0.00585 ± 0.00179	0.00149 ± 0.00062	0.0113 ± 0.00346
Transitions/Transversions	0	3/1	4/0	3/2

Notes. – Samples were divided into two populations for comparison: > 10,000 yr old (Pleistocene) and < 10,000 yr old (Holocene).

**Table 5:** Summary of variable sites for 1,180 bp of cyt *b *sequence, OMTai39 compared to 5 modern muskox

**Sample**	**37**	**85**	**136**	**174**	**213**	**246**	**324**	**345**	**365**	**435**
SH-OMMod01	T	T	T	C	G	A	T	A	T	C
SH-OMMod02	.	G	.	.	.	.	.	.	.	.
SH-OMMod03	.	G	.	.	.	G	.	.	.	.
SH-OMMod04	A	.	.	.	.	.	.	.	.	.
SH-OMMod05	.	.	C	T	.	.	.	.	.	.
*EH-OMTai39*	.	.	.	.	*A*	.	*C*	*C*	*C*	*T*

Notes. – Sample names and symbols as in table 2. Positions numbered from A of ATG start codon. Extinct haplotype italicized.

This example represents a relatively extreme case, as most PCRs yielded identical or nearly identical sequences that differed by minor among-clone variations that can be explained by DNA polymerase/sequencing errors or DNA damage in the templates (for example, note the clone sequences for OMTai39 in additional fig. 2). Where a position or several positions were not determinable after two independent amplifications, additional PCRs were performed, clones sequenced, and the majority base scored as the bona fide mtDNA sequence for each position of each sequence. In other words, each base of each sequence was covered by multiple PCRs and multiple clone sequences to determine the majority base for a given position as in Krings *et al. *[12].

In addition, six other samples (OMTai95, OMWra2, OMTai31670, OMYuk02, OMArA09, and OMYak12) yielded some sequence information, but the amount of DNA recovered was insufficient to permit two independent replications for each fragment and/or retrieve all four fragments of interest in this study (see table 1).

Overlapping PCR reactions were used to identify possible Numts and to prevent mischaracterizing Numts as organellar mtDNA [14,23]. In some species DNA extracted from hair may yield Numts at a higher frequency than do other tissues [24], making it a useful proxy for gauging the possibility of Numt contamination. A hair sample (OMModCl, table 1) from a living muskox was amplified, products cloned, and multiple sequences determined using several of the primer combinations used in this study. Sequences were consistent in the overlapping regions and matched DNA from other modern muskoxen, indicating that the primers used in this study do not favor Numts over organellar mtDNA (additional fig. 1, 2).

### Cytochrome b

A 114-bp segment of cyt *b *was successfully retrieved from most of the working fossil samples (table 3); in three cases (OMTai14, 39, and 46) it was possible to amplify a 376 bp fragment. All specimens identified as SHs that yielded cyt *b *sequence were identical. Only partial sequence data could be recovered for OMWra2 and OMYak12 (table 3). However, for comparable runs of sequence, these specimens were identical to all other SHs.

Owing to institutional sampling restrictions and the low quality of retrieved DNA, analysis of OMTai95 had to be based on one repetition of each of the two overlapping fragments. Although results for this specimen are therefore tentative, its sequence does not differ from that of OMTai38 and OMTai 46 (not shown). Thus, despite some individual variation, in all cases samples could be classed unambiguously as either EH or SH.

Specimens in the EH group differed uniformly from those classed as SH by three fixed differences (table 3, positions 324, 346, and 366). There were also some internal differences within the EH group. OMTai14 and OMTai39 possessed an additional base substitution not seen in other members (position 436), and OMBolL15564 expressed a unique A to G substitution at position 425. Given the difficulties encountered in determining the mitochondrial HV region sequence for this last sample, this result was excluded from analysis.

The Holocene samples (*N *= 43) exhibited no variations. Only one haplotype was retrieved (table 4), which is consistent with Groves and Shields' [8] evidence for limited among-individual variation at the cyt *b *locus in living muskoxen. By contrast, the Pleistocene samples yielded 2 haplotypes among 4 sequences and thus greater among-individual haplotype and nucleotide diversity in a much smaller sample.

**Figure 4:**
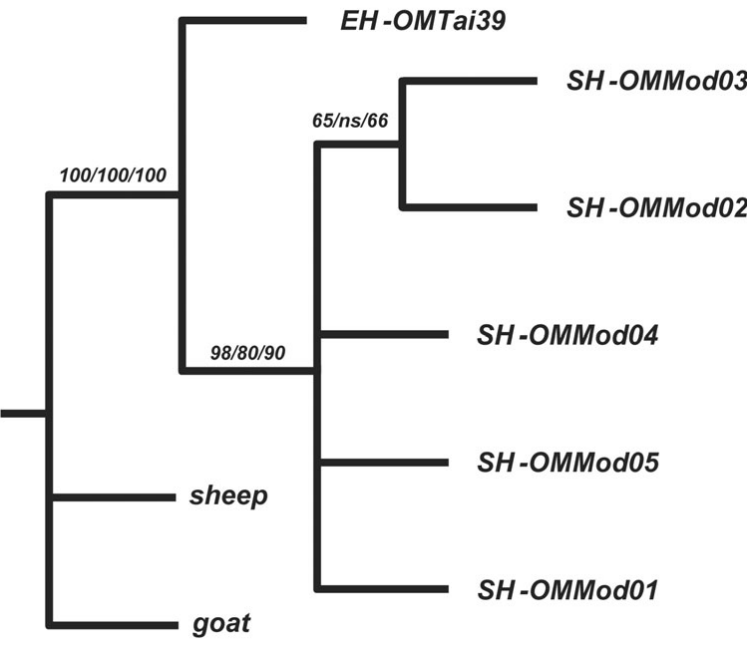
**Cyt *b *(1,180 bp) from a representative EH specimen (OMTai39) compared to modern *Ovibos moschatus *(OMMod01-05)**. Maximum parsimony tree using goat and sheep as outgroups. Calculated bootstrap support is shown for parsimony/neighbor joining/maximum likelihood at each node. Although analysis returned an evaluation of "ns" (no statistical support) for a given grouping in only one instance, significance of results should not be overemphasized. Nevertheless, it is parsimonious to conclude that (1) all muskoxen form a cluster as against sheep and goat, and (2) moderns form a structureless subcluster as against the extinct haplotype represented by OMTai39.

As a proxy for resolving the magnitude of differences among cyt *b *haplotypes, 1,180 bp of sequence in overlapping fragments was determined for one of the best-performing specimens, OMTai39 (table 5) and aligned by eye against 5 modern muskox cyt *b *sequences deposited in GenBank (accession numbers U17862 and U90300-U90303, here designated as OMMod01-05). Among modern muskoxen, within-group differences in cyt *b *sequences range from 1 to 4 base substitutions (table 5). By comparison, OMTai39 differed from all modern samples by 5–7 substitutions. Domestic sheep (*Ovis aries*) [GenBank: NC001941] and goat (*Capra hircus*) [GenBank: NC005044] were used as outgroups for phylogenetic analysis. The takin *Budorcas taxicolor *was not included in the analysis as Groves and Shields [8] have shown that the relationship between this species and muskox is not demonstrably closer than that of other caprines. When submitted to phylogenetic testing using maximum parsimony, neighbor joining, and maximum likelihood methods, OMTai39 placed in all analyses outside modern muskoxen cyt *b *sequences (bootstrap support, 80–98%) supporting the distinctiveness of Pleistocene vs. Holocene haplotypes (fig. 4).

### Hypervariable region

Samples from Yukon and islands in the Canadian Arctic Archipelago (OMYuk01, OMArA03, OMArA04, and OMArA05) displayed minor haplotype variation affecting 2–4 positions in the studied portion of the hypervariable region (additional fig. 1). No fixed differences were found between these samples and sequence data on modern muskoxen in GenBank [7](cf. OMMod01-08, OMModCl, table 5).

One position in the GenBank samples was not observed in any of the fossil sequences, nor was it found in a modern muskox sequence (OMModC1) that was acquired specifically to test results from this study (table 6, position 168). Because all of the sequences presented here are consensus results, generated from multiple PCRs and multiple clones sequenced on both strands, the difference in the database for modern samples is best interpreted as a sequencing artifact in the published sequences.

**Table 6:** Summary of variable positions in the hypervariable region of modern and ancient muskoxen

Source	**Sample**	**2**	**104**	**144**	**145**	**148**	**166**	**168**	**178**
Modern	SH-OMMod01	C	C	G	C	C	T	C	T
	SH-OMMod02	.	.	.	.	.	.	.	.
	SH-OMMod03	.	.	.	.	.	.	T	.
	SH-OMMod04	.	.	.	.	.	.	T	C
	SH-OMMod05	.	.	.	.	.	.	.	.
	SH-OMMod06	.	.	.	.	.	.	.	.
	SH-OMMod07	.	.	.	.	.	.	T	.
	SH-OMMod08	.	.	.	.	.	.	T	.
	SH-OMModCl	.	.	A	.	.	.	-	.
New World	SH-Yuk01	.	.	A	T	.	.	-	.
	SH-OMArA03	.	.	.	.	.	.	-	.
	SH-OMArA04	.	.	.	.	.	.	-	.
	SH-OMArA05	.	.	.	.	.	.	-	.
Old World	SH-OMWra2	nd	.	.	.	.	.	-	.
	SH-OMYak12	nd	.	.	.	.	.	-	.
	SH-OMTai23658.A	.	.	.	.	.	.	-	.
	SH-OMTai23658.B	.	.	A	.	.	.	-	.
	*EH-OMTai14*	*G*	*T*	*A*	.	.	.	-	.
	*EH-OMTai38*	.	*T*	*A*	.	*T*	*A*	-	.
	*EH-OMTai39*	*G*	*T*	*A*	.	.	.	-	.
	*EH-OMTai46*	.	*T*	*A*	.	*T*	*A*	-	.

Notes. – Sample names from table 1. Base positions numbered from first readable base from 5' amplified fragment. Identity to first sequence designated by a dot; differences indicated by base sequence. Deletions indicated by "-". For some samples, specific bases were not covered; these are designated "nd" (not determined). [Accession numbers for modern sequences are GenBank: NC005044, U47063, U47065, U47067, U47069, U47071, U47073, U47075.] Extinct haplotypes as determined for cyt *b *and HV region are italicized.

OMTai23658, one of two Holocene samples from Taimyr, yielded two unique haplotypes for the hypervariable region, identified here as A and B (table 6, position 144). Multiple PCRs, cloning, and sequencing of clones by two independent laboratories established that these haplotypes differ by 1 base, the difference likely being due to heteroplasmy (additional fig. 1; see also [26]). Haplotype A differed by 0–2 positions and haplotype B by 1–3 positions from sequences recovered for other fossil muskoxen from the Canadian Arctic Archipelago. The difference between both haplotypes of OMTai23658 and modern muskoxen was 1–3 substitutions.

All of the other successfully sequenced samples from Taimyr (OMTai14, OMTai38, OMTai39, and OMTai46) are of Pleistocene age (> 10,000 years ago). They were found to differ by 0–3 substitutions from one another but by 2–6 positions from modern muskoxen and the Canadian fossil samples. In addition, all the Pleistocene Taimyr samples presented 1 fixed difference not observed in any of the other samples studied (table 6, position 104), while OMTai38 and OMTai46 shared 2 base changes not found in any other specimen group (table 6, positions 148 and 166). OMTai14 and OMTai39 shared one position not found in any other specimen group (table 6, position 2).

The DNA yield from one other sample, OMBolL15564 from the New Siberian Islands, was low. However, it yielded reproducible EH sequence for cyt *b*. This is consistent with the sample's origin (near Taimyr) and age (late Pleistocene). Unfortunately, the HV region yielded several different sequences per amplification, including sequences that were similar to SH sequences, EH sequences, and ones that diverge from all others encountered in the course of this study (additional fig. 4). Since results for this specimen were highly inconsistent, we suspect some form of sample contamination but cannot identify the likely source. This required that we remove it form further analysis.

As may be seen in table 4, the Pleistocene sequences had more transversions (2 of 5 mutations) and slightly higher haplotype and nucleotide diversity per site measured than did Holocene sequences. However, given the almost 9-fold higher representation of Holocene compared to Pleistocene sequences, the statistics should be treated with caution. Higher among-sequence diversity (i.e., 2 haplotypes from 4 sequences as opposed to 4 from 44) for Holocene samples supports the concept that genetic diversity in muskoxen has been restricted. This is also consistent with the results obtained for cyt *b *(table 4).

**Figure 5:**
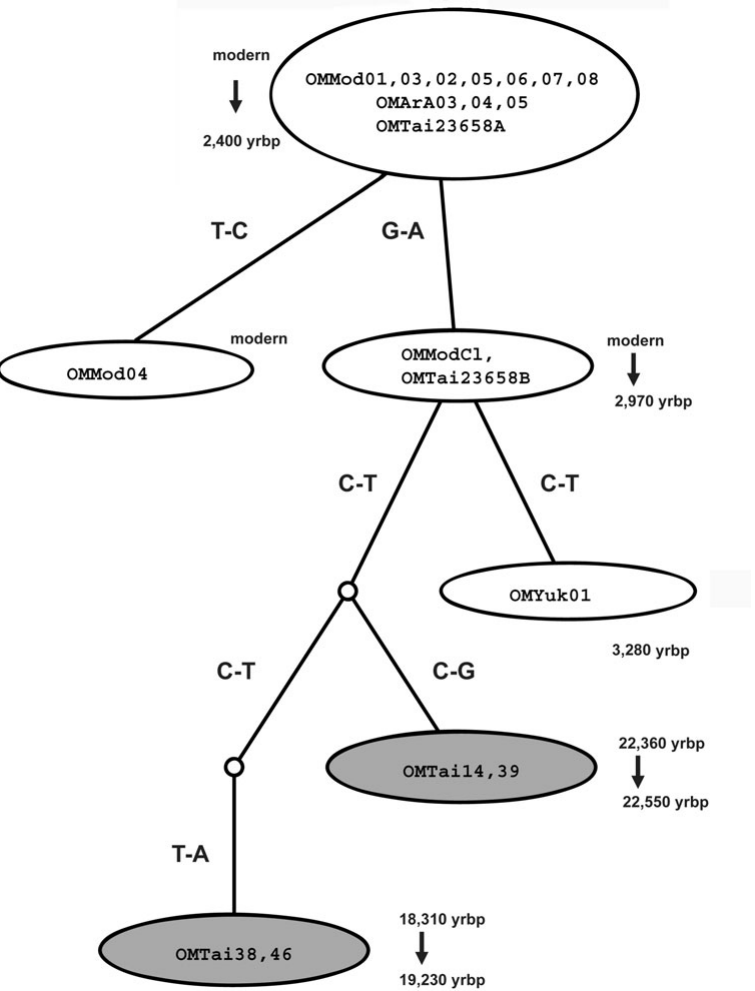
**Minimum spanning network for hypervariable region sequences**. The network is derived from the alignment shown in table 6; though indicated, the indel was omitted as it is a likely sequencing artifact in the database. Identical sequences are grouped together; EH samples are indicated by shading. The mutational change and sample mean radiocarbon age ranges (in yrbp) are shown next to each branch.

To further clarify among-sequence relationships, we depict the sequence network suggested by our data in figure 5: nodes reflect missing intermediates and correlate with the number of mutational steps between sequences. As expected, all haplotypes of the SH group are very closely related. Most of the extinct haplotypes recovered differ somewhat among themselves, and all Taimyr EHs differ more substantially from SHs than do SHs among themselves. The distance between the extinct haplotypes and the closest SH sample is two to three mutational steps. The network supports the distinctiveness of most of the HV haplotypes of Pleistocene age.

## Discussion

In view of the small number of specimens analyzed, conclusions have to be tentative. The conclusion best supported by the data is that, compared to modern muskoxen, late Quaternary *Ovibos *populations were less homogeneous for the loci sampled in this investigation. In particular, one group of haplotypes (EHs) occurring in fossil specimens from northeastern Asia differs from haplotypes in existing databases (SHs). The actual number of differences is not great (see Results), but their existence is thrown into sharp relief by the virtual lack of variation in the modern wildherd. In view of the multiple safeguards utilized to insure accuracy and repeatability (extraction of several samples in independent laboratories, sequencing of multiple independent clones from two or more amplifications, using overlapping PCR fragments to determine sequences, and rejection of suspect or ambiguous results), we feel that we can exclude laboratory artifacts or DNA damage as the source of these differences.

When the extinct haplotypes might have disappeared is not known. In our sample, the latest accepted record (of 5) is 18,310 ± 70 yrbp (radiocarbon years before present), which tells us that populations in which these haplotypes occurred survived at least into the Late Glacial Maximum, but not whether they lasted until the period of intensified megafaunal extinction at the end of the Pleistocene approximately 10,000 years ago (although in view of the extensive end-Pleistocene radiocarbon record for *Ovibos *in Asia [32], we regard this as more likely than not).

Another conclusion that is supported by the limited evidence currently available is that the SH group was present in mid-Holocene Asian muskoxen. This inference is based on the fact that OMTai23658 from Taimyr yielded sequence data indistinguishable from modern. (We had less success with the only other Eurasian Holocene sample available to us, OMTai31670. For the sequences covered it was initially found to be identical to modern muskox, but repeated efforts to replicate results were unsuccessful.) Given available data, however, it is premature to conclude that EHs were definitely *not *present as late as the middle Holocene. If in the future other specimens of Holocene age come to light in Asia, it would be well worthwhile to sample them for aDNA studies.

A last point of interest is that *Ovibos*, one of the few high-latitude megafaunal mammals to have survived into recent times, has clearly done so with reduced genetic variability. As already noted, at what point before the present this variability was lost cannot be satisfactorily established with existing data. For species for which aDNA sequence information is substantially better, such as bears and bison, there is molecular evidence for repeated loss and turnover of haplotype diversity during the late Quaternary (cf. [19,20,27]). Although no aDNA investigation of the giant Irish deer, *Megaloceros*, has yet been published, Stuart *et al. *[28] have recently shown that the radiocarbon record for this taxon reveals dramatic changes in population distributions during the late Pleistocene, followed ultimately by total collapse in the mid-Holocene. Thus, the data presented here, in combination with studies performed on other megafaunal species, is consistent with a scenario in which genetic diversity in muskoxen was reduced prior to the Pleistocene/Holocene transition.

It would also be of great interest to know whether late Quaternary small mammals (few of which became extinct) were affected by population crashes at the same time as larger species were. Extensive work on the mitochondrial genetics and phylogeography of extant lemmings by Fedorov and coworkers [29,30] suggests that, in these rodents at least, bottlenecking and catastrophic loss of genetic diversity did not occur in late glacial/postglacial time. The robustness of this inference could be tested with aDNA methods similar to the ones utilized here, as there are large samples of reasonably well-dated fossil lemming material in the paleontological collections of various museums (cf. [31]).

## Conclusion

In summary, the combined genetic and chronometric evidence presented in this paper establishes that *Ovibos moschatus *was genetically more diverse in the late Pleistocene than it is today. Precisely when haplotypes not found in extant muskoxen were lost, and under what conditions, are matters that cannot be well constrained at present. However, the data decisively show that now-extinct haplotypes persisted in Asia to a point only a few thousand years earlier than the accepted onset of the end-Pleistocene extinctions. It is reasonable to predict that, with better sampling, it may eventually be possible to show that populations of *Ovibos moschatus*, far from passing through this period of heightened extinction unscathed, were in fact brought down to small numbers over much of their original range, perhaps almost to the vanishing point [32]. Why muskoxen should have survived nevertheless in these circumstances, while many other high-latitude species did not, is yet another puzzle within the overarching mystery of late Quaternary extinctions.

## Methods

### Samples

Despite their wide holarctic distribution during the late Quaternary, ovibovines are comparatively rare as fossils except in those few areas (e.g., Taimyr Peninsula, parts of Yukon; fig. 1) where conditions were evidently optimal for them during much of the late Pleistocene. We obtained our specimens from two broad areas, designated "New World" and "Old World" (respectively, northern North America including the Arctic Archipelago, and the Arctic periphery of Asia east of the Kara Sea). A total of 31 muskoxen samples were subjected to DNA extraction and PCR amplification using various primer combinations. Of these, 16 samples worked with varying degrees of success; table 1 and fig. 3 provide information on these specimens regarding locality, age, and other relevant data (see also additional figs. 1, 2, 3, 4). The 15 remaining samples, from Alaska, Russia, and the Canadian Arctic Archipelago, did not produce amplifiable DNA on extraction and are not shown. It is particularly unfortunate that none of the Alaskan material yielded DNA. There may be an age effect: Mateus *et al. *[33] showed that muskoxen were common in Alaska earlier in the Pleistocene, but became very rare after 40,000 yrbp (which is the upper practical limit of radiocarbon dating). Designations combine taxon acronym/geographical origin/sample number: thus OMArA05 stands for *Ovibos moschatus*/Arctic Archipelago/sample 5.

### Radiocarbon dating record

All specimens listed in table 1 had either been subsampled for ^14^C dating as part of this study or had been dated previously by other workers. Age estimates are cited in radiocarbon years before present without δ^13^C correction. All Beta Analytic estimates are accelerator dates on the collagen fraction of submitted specimens and typically have low associated standard errors. Other dates quoted in the table are based on older liquid scintillation technology which required large samples. However, accuracy and precision are not necessarily poorer in the latter case: OMTai23658 has been dated by both methods, and mean age estimates are statistically indistinguishable at 2 sigmas (table 1).

All samples yielded ^13^C/^12^C ratios within normal ranges for the materials dated (usually bone), and no difficulties with any samples were reported by the radiocarbon laboratory. Radiocarbon age estimates in excess of 40,000 yrbp are generally considered to be of questionable accuracy, and specimens so dated could be considerably older [37]. Although EH samples are generally older than SH samples, there is overlap around the time of the Last Glacial Maximum 21,000 calendar years ago [38], indicating that haplotype groups were coeval. Greater temporal overlap would probably have been found had sample sizes been larger.

Fig. 2 collates published finite ^14^C records for the study areas in the Old and New Worlds. Note that the distribution of dates in the New World record is quite asymmetrical, with few early dates and a heavy concentration in the late Holocene. A similar asymmetry is seen in fig. 3, which depicts the date distribution of specimens used in this study. The explanation concerns substantial differences in the amount of habitable range available to muskoxen during the period covered by radiocarbon dating. Because almost all of Canada and Greenland were covered by icecaps in the last (late Wisconsinan) cold phase, few early muskox dates would be expected from this area. Following icecap retreat, the muskoxen record increases markedly, especially after the mid-Holocene with return of a significant biomass of vascular plants at high latitudes [39]. By contrast, the Arctic periphery of Asia was not significantly glaciated, and muskox were evidently present throughout this zone during the latest Pleistocene, but rare or absent thereafter until ~4,000 yrbp when they made a brief reapperance.

### DNA extraction, PCR, and sequencing

All fossil samples were collected by the first author using an electric drill with stainless steel "keyhole" coring drill bits that were cleaned and air dried after each use. Samples were placed in individual plastic containers, labeled, and stored while in the field in cool conditions and on return in a -20°C freezer in an isolated lab. Protocols for DNA extractions, PCR conditions, PCR reamplification, cloning strategy, colony PCR and sequencing, have been described in detail in Krings *et al. *[12] and Greenwood [34], with the variant that (for most samples) DNA extractions were conducted with NucleoSpin^® ^DNA-Trace Kits in combination with NucleoSpin^® ^funnel columns (Macherey & Nagel), Gene Clean Kit (Qbiogene), or GOLD Forensic DNA Kit (peqlab) according to manufacturer protocols and eluted in 55 to 60μl 10 mM Tris-HCl, pH 8.5. Reamplifications of PCR products when necessary were done either by direct amplification from of an aliquot of the first PCR or subsequent to gel extraction of the primary amplification band using a Qiagen Gel Extraction Kit. A buffer control was taken through each extraction procedure with no sample added and used as a PCR negative control in each PCR reaction to control for contamination. PCR primers, combinations used, and sequences are shown in table 2. Modern hair and blood DNA were prepared in a separate laboratory and never brought to laboratories conducting aDNA work so as to avoid potential contamination. Both hair and blood were prepared using Qiagen QIAamp DNA Mini Kits and PCR performed using Roche Expand Taq or Promega Taq polymerase according to manufacturer instructions. PCR conditions used were 30 cycles of 94°C 30 seconds, 50°C 30 seconds and 72°C 30 seconds. Products were cloned and clones sequenced as described in Greenwood (2002).

**Table 2:** Primers used in reconstructing cyt *b *and HV sequences

**Target**	**Primer combination**	**Primer sequence**	**Notes**
Main cyt *b *primers	L1 + H1	L1: CCTATACTACGGATCATACA H1: AAACTGCAGCCCCTCAGAATGATATTTGTCCTCA	
	L2 + H2	L2: GGGGGAATTCTTCTACTCA H2: GCTGAGAGGAGGTTAGTGA	
Additional cyt *b *primers	L3 + H3	L3: CGAAGCTTGATATGAAAAACCATCGTTG H3: GTAGAATTAGGCAGATACCT	*a*
	L4 + H4	L4: TCAAACATCTCATCATGATG H4: GTAGAAGAATTACCCCGATG	*a*
	L5 + H5	L5: CTCCCATTTATCATCGTAGC H5: TTGATCGTAGGATTGCGTAT	*a*
	L6 + H6	L6: TCACATTAAACCAGAGTGAT H6: GGCATTAGCACCAGGATGAT	*a*
	L2 + H7	H7: TGGATCCTGTTTCGTGGAGG3	*b*
	L7 + H1	L7: AAAAAGCTTCCATCCAACATCTCAGCATGATGAAA	*c*
Main HV region primers	L1 + H1	L1: AAAGGATCCTAAACTACTCCCTGAATT H1: AAAGGATCCATCATGCGTTGTTGCGT	
	L2 + H2	L2: AAAGAATTCCCAGTATCAAATCTACC H2: AAAGGATCCGCTTGTGTTACATATGTCT	
Additional HV region primers	L3 + H3	L3: CACCCAAAGCTGAAGTTCTA H3: GTTGTTGCGTGTGGAGTAGG	*d*

Notes. – For certain samples, some primer pairs were more successful than others. Additional primer pairs were applied to a few samples during the course of the study as noted below. The Numt test for sample OMModCl was performed using primer combinations: cyt *b *L1 + H1, L2 + H2, L7 + H1, HV region L1 + H1, L2 + H2. All primers are shown 5' to 3'.

^*a*^Primers used to amplify nearly full length cyt *b *from OMTai39.

^*b*^Additional primer combination used on OMTai38, 39 and 46.

^*c*^Additional primer combination used on OMTai14 and 46.

^*d*^Additional primer combination used on OMTai14, 39, and 46.

Negative water controls and in many cases mock extraction controls were included in experiments to control for contamination. Extractions, amplification, and sequencing for samples OMTai23658, OMTai39, OMTai46, OMYuk01, OMArA03, OMArA04, and OMArA05 were performed independently at SR&D and Medigenomix; in each case the same sequences were obtained. Each amplified region was covered at least twice if possible; multiple clones were sequenced to determine the correct sequence and to avoid mis-scoring errors due to DNA damage [35]. In the few cases in which available material was insufficient for double coverage, the sequence result is regarded as tentative. The number of replications for each PCR for each sample is indicated in table 1. Sequences were also derived from overlapping PCR fragments as previously described [12] (see additional fig. 1, 2, 3). [All consensus sequences have been deposited with the following accession numbers in GenBank: AY839538-AY839551 (cyt *b*); AY839552-AY839563 (hypervariable region); and AY83956 (the only substantially complete cyt *b *sequence retrieved in this study for OMTai39).]

### Phylogenetic analysis

Maximum parsimony, neighbor joining, and maximum likelihood analyses were executed using PAUP 4.0b10 [36] with *Ovis *(sheep) and *Capra *(goat) as outgroups for the nearly-complete cyt *b *sequence of OMTai39 (fig. 4). Although 100 bootstrap replicates were performed for each tree generated, the limited variability present in the data requires that no great weight be placed on the results of such experiments (i.e., chances are high that the few variable sites will be included in the bootstrap and will therefore nearly always replicate the same tree). Nevertheless, a strictly parsimonious interpretation of the distribution of certain fixed differences (see text) indicates that sequence data can be used to diagnostically distinguish extinct from surviving haplotypes. Networks for the hypervariable region sequences derived from the alignment shown in table 6 were drawn by hand (fig. 5).

## Authors' contributions

RDEM participated in the design of the study, coordinated the drafting of the paper with the other authors, acquired and interpreted radiocarbon dates, and helped with phylogenetic analysis; ANT participated in the drafting of the paper and provided data and information from Russian sources; DM managed acquisition of specimens for sequence analysis in Russia and Canada, and participated in the drafting of the paper; and ADG participated in the design of the study, oversaw all lab work, sequence alignment, and phylogenetic analysis, and participated in drafting the paper. All authors read and approved the final manuscript.

## Supplementary Material

Additional File 1 **Alignment of hypervariable region clones from overlapping PCR fragments used to determine the sequences for each specimen analyzed**. The consensus sequence of OMModCl was used as a reference. Dots indicate identity to the consensus sequence. Differences are indicated. Base pair sequences that could not be determined for a given clone are marked "N". Gaps are indicated by "-". The first clone provides the name of each sample according to the labels provided in table 1. The numbers indicate PCR replication + clone number: thus 1.1 indicates PCR 1, first clone sequence. Click here for file

Additional File 2 **Alignment of cyt *b*clones from overlapping PCR fragments used to determine the sequences for each specimen analyzed**. Symbols as in additional fig. 1. Click here for file

Additional File 3 **Alignment of clones from overlapping PCR fragments used to determine 1,180 bp of OMTai39 cyt *b***. Symbols as in additional fig. 1. The reference is a modern muskox sequence taken from GenBank. Four additional modern muskox sequences are included in the alignment. Click here for file

Additional File 4 **Clone sequences derived from multiple amplifications of sample OMBolL15564**. All symbols are the same as in additional fig. 1, 2, 3. The modern DNA consensus sequence from additional fig. 1 is used as a reference sequence. Click here for file
