# Supplementary material for: Late Quaternary loss of genetic diversity in muskox (Ovibos)
Source: BMC Evol Biol. 2005 Oct 6;5:49. doi: 10.1186/1471-2148-5-49 (PMC1266356; doi:10.1186/1471-2148-5-49)
Supplement: Additional File 1 — Alignment of hypervariable region clones from overlapping PCR fragments used to determine the sequences for each specimen analyzed. The consensus sequence of OMModCl was used as a reference. Dots indicate identity to the consensus sequence. Differences are indicated. Base pair sequences that could not be determined for a given clone are marked "N". Gaps are indicated by "-". The first clone provides the name of each sample according to the labels provided in table 1. The numbers indicate PCR replication + clone number: thus 1.1 indicates PCR 1, first clone sequence. [file 1471-2148-5-49-S1.pdf]

```
                        |    |    |    |    |    |    |    |    |    |    |    |    |    |    |    |    |    |
                        5         15         25         35         45         55         65         75         85
OMModCl.Consensus   ACCACTAACA CCTTCTATTA ATATACCACT AAAAACATTA AGAGCCTTCC CAGTATCAAA TCTACCAAAA CTTTCAATAA TATAACACTA
OMModCl.1.1         .......... .......... .......... .......... .......... .......... .......... .......... ..........
1.2                 .......... .......... .......... .......C.. .......... .......... .......... .......... ..........
1.3                 .......... .......... .......... .......... .......... .......... .......... .......... ..........
1.1                                                                                       .... .......... ..........
1.2                                                                                       .... .......... ..........
1.3                                                                                       .... .......... ..........
OMYuk01.1.1         .......... .......... .......... .......... .......... .......... .......... .......... ..........
1.2                 .......... .......... .......... .......... .......... .......... .......... .......... ..........
1.3                 .......... .......... .......... .......... .......... .......... .......... .......... ..........
1.5                 .......... .......... .......... .......... .......... .......... .......... .......... ..........
2.1                 .......... .......... .......... .......... .......... .......... .......... .......... ..........
2.2                 .......... .......... .......... .......... .......... .......... .......... .......... ..........
2.3                 .......... .......... .......... .......... .......... .......... .......... .......... ..........
2.4                 .......... .......... .......... .....T.... .......... .......... .......... .......... ..........
1.1                                                                                       .... .......... ..........
2.1                                                                                       .... .......... ..........
2.2                                                                                       .... .......... ..........
2.3                                                                                       .... .......... ..........
2.4                                                                                       .... .......... ..........
OMArA03.1.1         .N........ .......... .......... ..G....... .......... .........G .......... .......... ..........
1.2                 .T........ .......... .......... .......... .......... .......... .......... .......... ..........
1.3                 .......... .......... .......... .......... .......... .......... .......... .......... ....G.....
1.4                 .......... .......... .......... .......... .......C.. .......... .......... ..C....... ....G.....
1.5                 .......... .......... .......... .......... .......... .......... .......... .......... ..........
2.1                 .......... .T........ .....T..T. .......... .......... .......... .......... .......... .....T....
2.2                 .......... .......... .......... .......... .......... .......... .......... .......... ..........
1.1                                                                                       .... .......... ..........
1.2                                                                                       .... .......... ..........
1.3                                                                                       .... .......... ..........
1.4                                                                                       .... .......... ..........
1.5                                                                                       .... .......... ..........
2.1                                                                                       .... .......... ..........
2.2                                                                                       .... .......... ..........
2.3                                                                                       .... .......... .......T..
OMArA04.1.1         .......... .......... .......... .......... .......... .......... .......... .......... ..........
1.2                 .......... .......... .......... .......... .......... .......... .......... .......... ..........
1.3                 .......... .......... .......... .......... .......... .......... .......N.. .......... ..........
1.4                 .......... .......... .......... .......... .......... .......... .......... .......... ..........
1.5                 .......... .......... .......... .......... .......... .......... .......... .......... ..........
2.1                 .......... .......... .......... .......... .......... .......... .......... .......... ..........
2.2                 .......... .......... .......... .......... .......... .......... .......... .......... ..........
1.1                                                                                       .... .......... ..........
1.2                                                                                       .... .......... ..........
1.3                                                                                       .... .......... ..........
1.4                                                                                       .... .......... ..........
2.1                                                                                       .... .......... ..........
2.2                                                                                       .... .......... ..........
2.3                                                                                       .... .......... ..........
2.4                                                                                       .... .......... ..........
2.5                                                                                       .... .......... ..........
OMArA05.1.1         .......... .......... .......... .......... .........T .......... .......... .......... ..........
1.2                 .......... .......... .......... .......... .......... .......... .......... .......... ..........
1.3                 .......... .......... .......... .......... .......... .......... .......... .......... ..........
1.4                 .......... .......... .......... .......... .......... .......... .......... .......... ..........
1.5                 .......... .......... .......... .......... .......... .......... .......... .......... ..........
2.1                 .......... .......... .......... .......... .......... .......... .......... .......... ..........
2.2                 .......... .......... .......... .......... .......... .......... .......... .......... ..........
2.3                 .......... .......... .......... .......... .......... .......... .......... .......... ..........
2.4                 .......... .......... .......... .......... .......... .......... .......... .......... ..........
1.1                                                                                       .... .......... ..........
1.2                                                                                       .... .......... ..........
1.3                                                                                       .... .......... ..........
1.4                                                                                       .... .......... ..........
1.5                                                                                       .... .......... ..........
2.1                                                                                       .... .......... ..........
2.2                                                                                       .... .......... ..........
2.3                                                                                       .... .......... ..........
2.4                                                                                       .... .......... ..........
2.5                                                                                       .... .......... ..........
OMWra02.1.1                                                                               .... .......... ..........
1.2                                                                                       .... .......... ..........
1.3                                                                                       .... .......... ..........
1.4                                                                                       .... .......... ..........
1.5                                                                                       .... .......... N.........
2.1                                                                                       .... .......... ..........
2.2                                                                                       .... .......... ..........
2.3                                                                                       .... .......... ..........
2.4                                                                                       .... .......... ..........
2.5                                                                                       .... .......... ..........
OMYak12.1.1                                                                               .... .......... ..........
1.2                                                                                       .... .......... ..........
1.3                                                                                       .... .......... ..........
1.4                                                                                       .... .......... ..........
1.5                                                                                       .... .......... ..........
2.1                                                                                       .... .C........ ..........
2.2                                                                                       .... .......... ..........
2.3                                                                                       .... .......... .........G
2.4                                                                                       .... .......... ..........
2.5                                                                                       .... .......... ..........
OMTai23658.1.1      .......... .......... .......... .......... .......... .......... .......... .......... ..........
1.2                 .......... .......... .......... .......... .......... .......... .......... .......... ..........
1.3                 .......... .......... .......... .......... .......... .......... .......... .......... ..........
1.5                 .......... .......... .......... .......... .......... .......... .......... .......... ..........
1.6                 ..A....... .......... .......... .......... .......... .......... .......... .......... ..........
2.1                 .......... .......... .......... .......... .......... .......... .......... .......... ..........
2.2                 .......... .......... .......... .......... .......... .......... .......... .......... ..........
1.1                                                                                       .... .......... ..........
1.2                                                                                       .... .......... ..........
1.3                                                                                       .... .......... ..........
1.4                                                                                       .... .......... ..........
1.5                                                                                       .... .......... ..........
2.1                                                                                       .... .......... ..........
2.2                                                                                       .... .......... ..........
2.3                                                                                       .... .......... ..........
2.4                                                                                       .... .......... ..........
2.5                                                                                       .... .......... ..........
3.1                                                                                       .... .......... ..........
3.2                                                                                       .... .......... ..........
3.3                                                                                       .... .......... ..........
3.4                                                                                       .... .......... ..........
3.5                                                                                       .... .......... ..........
4.1                                                                                       .... .......... ..........
4.2                                                                                       .... .......... ..........
4.3                                                                                       .... .......... ..........
4.4                                                                                       .... .......... ..........
4.5                                                                                       .... .......... ..........
5.1                                                                                       .... .......... ..........
5.2                                                                                       .... .......... ..........
5.3                                                                                       .... .......... ..........
5.4                                                                                       .... .......... ..........
5.5                                                                                       .... .......... ..........
OMTai14.1.1         .G........ .......... .......... .......... .......... .......... .......... .......... ..........
1.2                 .G........ .......... .......... .......... .......... .......... .......... .......... ..........
2.1                 .G........ .......... .......... .......... .......... .......... .......... .......... ..........
2.2                 .G........ .......... .......... .......... .......... .......... .......... .......... ..........
2.3                 .G........ .......... .......... .......... .......... .......... .......... .......... ..........
2.4                 .G........ .......... .......... .......... .......... .......... .......... .......... ..........
2.5                 .G........ .......... .......... .......... .......... .......... .......... .......... ..........
1.1                                                                                       .... .......... ..........
1.2                                                                                       .... .......... ..........
1.3                                                                                       .... .......... ..........
1.4                                                                                       .... .......... ..........
1.5                                                                                       .... .......... ..........
2.1                                                                                       .... .......... ..........
2.2                                                                                       .... .......... ..........
2.3                                                                                       .... .......... ..........
2.4                                                                                       .... .......... ..........
2.5                                                                                       .... .......... ..........
OMTai38.1.1         .......... .......... .......... .......... .......... .......... .......... .......... ..........
1.2                 .......... .......... .......... .......... .......... .......... .......... .......... ..........
1.3                 .......... .......... .......... .......... .......... .......... .......... .......... ..........
1.4                 .......... .......... .......... .......... .......... .......... .......... .......... ..........
1.5                 .......... .......... .......... .......... .......... .......... .......... .......... ..........
2.1                 .......... .......... .......... .......... .......... .......... .......... .......... ..........
2.2                 .......... .......... .......... .......... .......... .......... .......... .......... ..........
1.1                                                                                       .... .......... ..........
1.2                                                                                       .... .......... ..........
1.3                                                                                       .... .......... ..........
1.4                                                                                       .... .......... ..........
2.1                                                                                       .... .......... ..........
2.2                                                                                       .... .......... ..........
2.3                                                                                       .... .......... ..........
2.4                                                                                       .... .......... ..........
2.5                                                                                       .... .......... ..........
OMTai39.1.1         .G........ .......... .......... .......... .......... .......... .......... .......... ..........
2.1                 .G........ .......... .......... .......... .......... .......... .......... .......... ..........
2.2                 .G........ .......... .......... .......... .......... .......... .......... .......... ..........
2.3                 .G........ .......... .......... .......... .......... .......... .......... .......... ..........
2.4                 .G........ .......... .......... .......... .......... .......... .......... .......... ..........
2.5                 .G........ .......... .......... .......... .......... .......... .......... .......... ..........
1.1                                                                                       .... .......... ..........
1.2                                                                                       .... .......... ..........
1.3                                                                                       .... .......... ..........
1.4                                                                                       .... .......... ..........
1.5                                                                                       .... .......... ..........
2.1                                                                                       .... .......... ..........
2.2                                                                                       .... .......... ..........
2.3                                                                                       .... .......... ..........
2.4                                                                                       .... .......... ..........
2.5                                                                                       .... .......... ..........
OMTai46.1.1         .......... .......... .......... .......... .......... .......... .......... .......... ..........
1.2                 .......... .......... .......... .......... .......... .......... .......... .......... ..........
1.4                 .......... .......... .......... .......... .......... .......... .......... .......... ..........
1.5                 .......... .......... .......... .......... .......... .......... .......... .......... ..........
1.6                 .......... .......... .......... .......... .......... .......... .......... .......... ..........
2.1                 .......... .......... .......... .......... .......... .......... .......... .......... ..........

2.2                 .......... .......... .......... .......... .......... .......... .......... .......... ..........
2.3                 .......... .......... .......... .......... .......... .......... .......... .......... ..........
2.4                 .......... .......... .......... .......... .......... .......... .......... .......... ..........
2.5                 .......... .......... .......... .......... .......... .......... .......... .......... ..........
1.1                                                                                       .... .......... ..........
1.2                                                                                       .... .......... ..........
1.3                                                                                       .... .......... ..........
1.4                                                                                       .... .......... ..........
1.5                                                                                       .... .......... ..........
2.1                                                                                       .... .......... ..........
2.2                                                                                       .... .......... ..........
2.3                                                                                       .... .......... ..........
3.1                                                                                       .c.. .......... ..........
3.2                                                                                       .... .......... ..........
3.3                                                                                       .... .......... ..........
3.4                                                                                       .... .......... ..........
3.5                                                                                       .... .......... ..........

                        |    |    |    |    |    |    |    |    |    |    |    |    |    |    |    |    |
                        95        105        115        125        135        145        155        165        175
OMModCl.Consensus   ATTTCCTACT CCACACGCAA CAACGCATGA TGGACGTAAT ATATGTTTAG TACACAACCC ATAATCTTAA CCCATTAAAT AGCACGT
OMModCl.1.1         .......... ....
1.2                 .......... ....
1.3                 .......... ....
1.1                 .......... .......... .......... .......... .......... .......... .......... .......... .......
1.2                 .......... .......... .......... .......... .......... .......... .......... .......... .......
1.3                 .......... .......... .......... .......... .......... .......... .......... .......... .......
OMYuk01.1.1         .......... ....
1.2                 .......... ..CT
1.3                 .......... ....
1.5                 .......... ..CT
2.1                 .......... ....
2.2                 .......... ....
2.3                 .......... ....
2.4                 .......... ....
1.1                 .......... .......... .......... .......... .......... ....T..... .......... .......... .......
2.1                 .......... .......... .......... .......... .......... ....T..... .......... .......... G......
2.2                 .......... .......... .......... .......... .......... ....T..... .......... .......... .......
2.3                 .......... .......... .......... .......... .......... ....T..... .......... .......... .......
2.4                 .......... .......... .......... .......... .......... ....T..... .......... .......... .......
OMArA03.1.1         .......... ....
1.2                 .......... ....
1.3                 .......... ....
1.4                 .......... ....
1.5                 .......... ....
2.1                 .......... ....
2.2                 .......... ....
1.1                 .......... .......... .......... .......... .......... ...G...... .......... .......... .......
1.2                 .......... .......... .......... .......... .......... ...G...... .......... .......... .......
1.3                 .......... .......... .......... .......... .......... ...G...... .......... .......... .......
1.4                 .......... .......... .......... .......... .......... ...G...... .......... .......... .......
1.5                 .......... .......... .......... .......... .......... ...N...... .......... .......... .......
2.1                 .......... .......... .......... .......... .......... ...G...... .......... .......... .......
2.2                 .......... .......... .......... .......... .......... ...G...... .......... .......... .......
2.3                 .......... .......... .......... .......... .......... ...G...... .......... .......... .......
OMArA04.1.1         .......... ....
1.2                 .......... ....
1.3                 .......... ....
1.4                 .......... ....
1.5                 .......... ....
2.1                 .......... ....
2.2                 .......... ....
1.1                 .......... .......... .......... .......... .......... ...G...... .......... .......... .......
1.2                 .......... .......... .......... .......... .......... ...G...... .......... .......... .......
1.3                 .......... .......... .......... .......... .......... ...G...... .......... .......... .......
1.4                 .......... .......... .......... .......... .......... ...G...... .......... .......... .......
2.1                 .......... .......... .......... .......... .......... ...G...... .......... .......... .......
2.2                 .......... .......... .......... .......... .......... ...G...... .......... .......... .......
2.3                 .......... .......... .......... .......... .......... ....T..... .......... .......... .......
2.4                 .......... .......... .......... .......... .......... ...G...... .......... .......... .......
2.5                 .......... .......... .......... .......... ........G. ...G...... .......... .......... .......
OMArA05.1.1         .......... ....-----. .......... .......... .......... .......... .......... .......... .......
1.2                 .......... ....
1.3                 .......... ....
1.4                 .......... ....
1.5                 .......... ....
2.1                 .......... ....
2.2                 .......... ....
2.3                 .......... ....
2.4                 .......... ....
1.1                 .......... .......... ....A..... .......... .......... ...G...... .......... .......... .......
1.2                 .......... .......... .......... .......... .......... ...G...... .......... .......... .......
1.3                 .......... .......... .......... .......... .......... ...G...... .......... .......... .......
1.4                 .......... .......... .......... .......... .......... ...G...... .......... .......... .......
1.5                 .......... .......... .......... .......... .......... ...G...... .......... T......... .......
2.1                 .......... .......... .......... .......... .......... ...G...... .......... .......... .......
2.2                 .......... .......... .......... .......... .......... ...G...... .......... .......... .......
2.3                 .......... .......... .......... .......... .......... ...G...... .......... .......... .......
2.4                 .......... .......... .......... .......... .......... ...G...... .......... .......... .......
2.5                 .......... .......... .......... .......... .......... ...G...... .......... .......... .......
OMWra02.1.1         .......... .......... .......... .......... .......... ...G...... .......... .......... .......
1.2                 .......... .......... .......... .......... .......... ...G...... .......... .......... .......
1.3                 .......... .......... .......... .......... .......... ...G...... .......... .......... .......
1.4                 .......... .......... .......... .......... .......... ...G...... .......... .......... .......
1.5                 .......... .......... .......... .......... .......... ...G...... .......... .......... .......
2.1                 .......... .......... .......... .......... .......... ...G...... .......... .......... .......
2.2                 .......... .......... .......... .......... .......... ...G...... .......... .......... .......
2.3                 .......... .......... .......... .......... .......... ...G...... .......... .......... .......

2.4                 .......... .......... .......... .......... .......... ...G...... .......... .......... .......
2.5                 .......... .......... .......... .......... .......... ...G...... .......... .......... .......
OMYak12.1.1         .......... .......... .......... .......... .......... ...G...... .......... .......... .......
1.2                 .......... .......... .......... .......... .......... ...G...... .......... .......... .......
1.3                 .......... .......... .......... .......... .......... ...G...... .......... .......... .......
1.4                 .......... .......... .......... .......... .......... ...G...... .......... .......... .......
1.5                 .......... .......... .......... .......... .......... ...G...... .......... .......... .......
2.1                 .......... .......... .......... .......... .......... ...G...... .......... .......... .......
2.2                 .......... .......... .......... .......... .......... ...G...... .......... .......... .......
2.3                 .......... .......... .......... .......... .......... ...G...... .......... .......... .......
2.4                 .......... .......... .......... .......... .......... ...G...... G......... .......... .......
2.5                 .......... .......... .......... .......... .......... ...G...... .......... .......... .......
OMTai23658.1.1      .......... ....
1.2                 .......... ..CT
1.3                 .......... ....
1.5                 .......... ....
1.6                 .......... ....
2.1                 .......... ....
2.2                 .......... ....
1.1                 .......... .......... .......... .......... .......... ...G...... .......... .......... .......
1.2                 .......... .......... .......... .......... .......... ...G...... .......... .......... .......
1.3                 .......... .......... .......... .......... .......... ...G...... .......... .......... .......
1.4                 .......... .......... .......... .......... .......... ...G...... .......... .......... .......
1.5                 .......... .......... .......... .......... .......... ...G...... .......... .......... .......
2.1                 .......... .......... .......... .......... .......... .......... .......... .......... .......
2.2                 .......... .......... .......... .......... .......... .......... .......... .......... .......
2.3                 .......... .......... .......... .......... .......... .......... .......... .......... .......
2.4                 .......... .......... .......... .......... .......... .......... .......... .......... .......
2.5                 .......... .......... .......... .......... .......... .......... .......... .......... .......
3.1                 .......... .......... .......... .......... .......... ...G...... .......... .......... .......
3.2                 .......... .......... .......... .......... .......... ...G...... .......... .......... .......
3.3                 .......... .......... .......... .......... .......... ...G...... .......... .......... .......
3.4                 .......... .......... .......... .......... .......... ...G...... .......... .......... .......
3.5                 .......... .......... .......... .......... .......... ...G...... .......... .......... .......
4.1                 .......... .......... .......... .......... .......... ...G...... .......... .......... .......
4.2                 .......... .......... .......... .......... .......... ...G...... .......... .......... .......
4.3                 .......... .......... .......... .......... .......... ...G...... .......... .......... .......
4.4                 .......... .......... .......... .......... .......... ...G...... .......... .......... .......
4.5                 .......... .......... .......... .......... .......... ...G...... .......... .......... .......
5.1                 .......... .......... .......... .......... .......... .......... .......... .......... .......
5.2                 .......... .......... .......... .......... .......... .......... .......... .......... .......
5.3                 .......... .......... .......... .......... .......... .......... .......... .......... .......
5.4                 .......... .......... .......... .......... .......... .......... .......... .......... .......
5.5                 .......... .......... .......... .......... .......... .......... .......... .......... .......
OMTai14.1.1         ....
1.2                 ....
2.1                 .......... ...T
2.2                 .......... ...T
2.3                 .......... ..CT
2.4                 .......... ...T
2.5                 .......... ...T
1.1                 .......... ...T...... .......... .......... .......... .......... .......... .......... .......
1.2                 .......... ...T...... .......... .......... .......... .......... .......... .......... .......
1.3                 .......... ...T...... .......... .......... .......... .......... .......... .......... .......
1.4                 .......... ...T...... .......... .......... .......... .......... .......... .......... .......
1.5                 .......... ...T...... .......... .......... .......... .......... .......... .......... .......
2.1                 .......... ...T...... .......... .......... .......... .......... .......... .......... .......
2.2                 .......... ...T...... .......... .......... .......... .......... .......... .......... .......
2.3                 .......... ...T...... .......... .......... .......... .......... .......... .......... .......
2.4                 .......... ...T...... .......... .......... .......... .......... .......... .......... .......
2.5                 .......... ...T...... .......... .......... .......... .......... .......... .......... .......
OMTai38.1.1         .......... ...T
1.2                 .......... ...T
1.3                 .......... ...T
1.4                 .......... ...T
1.5                 .......... ...T
2.1                 .......... ...T
2.2                 .......... ...T
1.1                 .......... ...T...... .......... .......... .......... .......T.. .......... .....A.... .......
1.2                 .......... ...T...... .......... .......... .......... .......T.. .......... .....A.... .......
1.3                 .......... ...T...... .......... .......... .......... .......T.. .......... .....A.... .......
1.4                 .......... ...T...... .......... .......... .......... .......T.. .......... .....A.... .......
2.1                 .......... ...T...... .......... .......... .......... .......T.. .......... .....A.... .......
2.2                 .......... ...T...... .......... .......... .......... .......T.. .......... .....A.... .......
2.3                 .......... ...T...... .......... .......... .......... .......T.. .......... .....A.... .......
2.4                 .......... ...T...... .......... .......... .......... .......T.. .......... .....A.... .......
2.5                 .......... ...T...... .......... .......... .......... .......T.. .......... .....A.... .......
OMTai39.1.1         ....
2.1                 .......... ...T
2.2                 .......... ...T
2.3                 .......... ...T

2.4                 .......... ...T
2.5                 .......... ...T
1.1                 .......... ...T...... .......... .......... .......... .......... .......... .......... .......
1.2                 .......... ...T...... .......... .......... .......... .......... .......... .......... .......
1.3                 .......... ...T...... .......... .......... .......... .......... .......... .......... .......
1.4                 .......... A..T...... .......... .......... .......... .......... .......... .......... .......
1.5                 .......... ...T...... .......... .......... .......... .......... .......... .......... .......
2.1                 .......... ...T...... .......... .......... .......... .......... .......... .......... .......
2.2                 .......... ...T...... .......... .......... .......... .......... .......... .......... .......
2.3                 .......... ...T...... .......... .......... .......... .......... .......... .......... .......
2.4                 .......... ...T...... .......... .......... .......... .......... .......... .......... .......
2.5                 .......... ...T...... .......... .......... .......... .......... .......... .......... .......
OMTai46.1.1         ....
1.2                 ....
1.4                 ....
1.5                 ....
1.6                 ....
2.1                 .......... ...T
2.2                 .......... ...T
2.3                 .......... ...T
2.4                 .......... ...T
2.5                 .......... ...T
1.1                 .......... ...T...... .......... .......... .......... .......T.. .......... .....A.... .......
1.2                 .......... ...T...... .......... .......... .......... .......T.. .......... .....A.... .......
1.3                 .......... ...T...... .......... .......... .......... .......T.. .......... .....A.... .......
1.4                 .......... ...T...... .......... .......... .......... .......T.. .......... .....A.... .......
1.5                 .......... ...T...... .......... .......... .......... .......T.. .......... .....A.... .......
2.1                 .......... ...T...... .......... .......... .......... .......T.. .......... .....A.... .......
2.2                 .......... .......... .......... .......... .......... ...G...... .......... .......... .......
2.3                 .......... .......... .......... .......... .......... ...G...... .......... .......... .......
3.1                 .......... ...T...... .......... .......... .......... .......T.. .......... .....A.... .......
3.2                 .......... ...T...... .......... .......... .......... .......T.. .......... .....A.... .......
3.3                 .......... ...T...... .......... .......... .......... .......T.. .......... .....A.... .......
3.4                 .......... ...T...... .......... .......... .......... .......T.. .......... .....A.... .......
3.5                 .......... ...T...... .......... .......... .......... .......T.. .......... .....A.... .......
```
