# Supplementary material for: Late Quaternary loss of genetic diversity in muskox (Ovibos)
Source: BMC Evol Biol. 2005 Oct 6;5:49. doi: 10.1186/1471-2148-5-49 (PMC1266356; doi:10.1186/1471-2148-5-49)
Supplement: Additional File 4 — Clone sequences derived from multiple amplifications of sample OMBolL15564. All symbols are the same as in additional fig. 1, 2, 3. The modern DNA consensus sequence from additional fig. 1 is used as a reference sequence. [file 1471-2148-5-49-S4.pdf]

```
                          |    |     |    |     |    |     |    |     |    |     |    |     |    |     |    |     |    |
                          5          15         25         35         45         55         65         75         85
OMModCl.consensus     ACCACTAACA -CCTTCTATT AATATACCAC TAAAAACATT AAGAGCCTTC CCAGTATCAA ATCTACCAAA ACTTTCAATA ATATAACACT
OMTai38               .......... -......... .......... .......... .......... .......... .......... .......... ..........
OMTai14               .G........ -......... .......... .......... .......... .......... .......... .......... ..........
OMBol15564.1.1        ..T.T..... T.A.CTATCA .TATATT.C. C......... ........C. .......... ..TCG..... ........C. ..........
1.2                   .......... -......... .......... .......... .......... .......... .......... .......... ..........
1.3                   .......... -......... .......... .......... .......... .......... .......... .......... ..........
1.4                   .........G -......... .G...T.... C......... .......... .......... .......... .......... ..........
1.5                   ..T.T..... TTA.CTATCA .TATATT.C. C......... ........C. .......... ...CG..... ........C. ..........
2.1                   .......... -.T....... ......T..T .......... .......... .......... .......... .......... ......T...
2.2                   ..T.T..... TTA.CTATCA .TATATT.CT C......... ........C. .......... ...CG..... ........C. ..........
2.3                   .........G -......... .G...T.... C......... .......... .......... .......... .......... ..........
2.4                   .A..T.---- --A.CTATCA .TATATT.C. C......... ........C. .......... ...CG..... ........C. ..........
2.5                   .......... -......... .......... .......... .......... .......... .......... .......... ..........
3.1                   .......... -......... .......... .......... .......... .......... .......... .......... ..........
4.1                   .G........ -......... .......... .......... .......... .......... .......... .......... ..........
4.2                   .G........ -......... .......... .......... .......... .......... ....G..... .......... ..........
4.3                   .G........ -......... .......... .......... .......... .......... .......... .......... ..........
4.4                   .G........ -......... .......... .......... G....T.... .......... .......... .......... ..........
4.5                   .G........ -......... .......... .......... .......... .......... .......... .......... ..........
1.1                                                                                              ... .......... ..........
1.2                                                                                              ... .......... ..........
1.3                                                                                              ... .......... ..........
1.4                                                                                              ... .......... ..........
1.5                                                                                              ... .......... ..........
2.1                                                                                              ... .......... ..........
2.2                                                                                              ... .......... ..........
2.3                                                                                              ... .......... ..........
2.4                                                                                              ... .......... ..........
2.5                                                                                              ... .......... ..........
3.1                                                                                              ... .......... ..........
3.2                                                                                              ... .......... ..........
3.3                                                                                              ... .......... ..........
3.4                                                                                              ... .......... ..........
3.5                                                                                              ... .......... ..........

                          |    |     |    |     |    |     |    |     |    |     |    |     |    |     |    |     |
                          95         105        115        125        135        145        155        165        175
OMModCl.consensus     AATTTCCTAC TCCACACGCA ACAACGCATG ATGGACGTAA TATATGTTTA GTACACAACC CATAATCTTA ACCCATTAAA TAGCACGT
OMTai38               .......... ....T..... .......... .......... .......... ........T. .......... ......A... ........
OMTai14               .......... ....T..... .......... .......... .......... .......... .......... .......... ........
OMBol15564.1.1        ..C....C.. .....
1.2                   .......... .....
1.3                   .......... .....
1.4                   .......C.. .....
1.5                   ..C....C.. .....
2.1                   .......... .....
2.2                   ..C....C.. .....
2.3                   .......C.. .....
2.4                   ..C....C.. .....
2.5                   .......... .....
3.1                   .......... .....
4.1                   .......... ....T
4.2                   .......... ....T
4.3                   .......... ....T
4.4                   .......... ....T
4.5                   .......... ....T
1.1                   .......... .......... .......... .......... .......... ....G..... .......... .......... ........
1.2                   .......... .......... .......... .......... .......... ....G..... .......... .......... ........
1.3                   .......... .......... .......... .......... .......... ....G..... .......... .......... ........
1.4                   .......... .......... .......... .......... .......... ....G..... .......... .......... ........
1.5                   .......... .......... .......... .......... .......... ....G..... .......... .......... ........
2.1                   .......... .......... .......... .......... .......... ....G..... .......... .......... ........
2.2                   .......... .......... .......... .......... .......... ....G..... .......... .......... ........
2.3                   .......... .......... .......... .......... .......... ....G..... .......... .......... ........
2.4                   .......... .......... .......... .......... .......... ....G..... .......... .......... ........
2.5                   .......... .......... .......... .......... .......... ....G..... .......... .......... ........
3.1                   ....C..... ....T..... .......... .......... .......... .......... .......... .......... ........
3.2                   .......... ....T..... .......... .......... .......... .......... .......... .......... ........
3.3                   .......... ....T..... .......... .......... .......... .......... .......... .......... ........
3.4                   .......... ....T..... .......... .......... .......... .......... .......... .......... ........
3.5                   .......... ....T..... .......... .......... .......... .......... .......... .......... ........
```
